# Expansion of Necrosis Depending on Hybrid Motor-Driven Motility of *Aeromonas hydrophila* in a Murine Wound Infection Model

**DOI:** 10.3390/microorganisms9010010

**Published:** 2020-12-22

**Authors:** Kohei Yamazaki, Takashige Kashimoto, Ayuha Niwano, Moeko Yamasaki, Mayu Nomura, Yukihiro Akeda, Shunji Ueno

**Affiliations:** 1 Laboratory of Veterinary Public Health, School of Veterinary Medicine, Kitasato University, 23-35-1, Higashi, Towada Aomori 034-8628, Japan; kyamazak@vmas.kitasato-u.ac.jp (K.Y.); twd_ayu@yahoo.co.jp (A.N.); moekoyama5@biken.osaka-u.ac.jp (M.Y.); vm15101n@st.kitasato-u.ac.jp (M.N.); ueno@vmas.kitasato-u.ac.jp (S.U.); 2 Division of Infection Control and Prevention, Osaka University Hospital, 2-2, Yamadaoka, Suita, Osaka 565-0871, Japan; akeda@biken.osaka-u.ac.jp

**Keywords:** *Aeromonas hydrophila*, necrotic soft tissue infection, motility, flagellum

## Abstract

The gram-negative bacterium *Aeromonas hydrophila* is a cause of fulminant and lethal necrotizing soft tissue infections (NSTIs). Suppressing the rapid proliferation of the pathogen and expansion of the necrosis caused in the host is an important issue in clinical practice, but the pathogenic mechanism for the rapid aggravation has not been clarified. In this study, we characterized the function of two types of motor stators in *A. hydrophila* and explored the role of motility during wound infection. In vitro analysis showed that the motility was reliably maintained while being complemented by the stators. We created a non-motile strain that lacked genes encoding two types of motor stators and analyzed the role of motility in a murine wound infection model. Examination of the bacterial burden in the local infection site and systemic circulation revealed that motility was not essential for the proliferation of *A. hydrophila* in the host. However, the extent of necrosis at the lesions was lower, and survival times were prolonged in mice infected with the non-motile strain compared with mice infected with the parent strain. These results provide evidence that the rapid expansion of necrosis and the progression to death within a short time period is dependent on the motility of *A. hydrophila*.

## 1. Introduction

*Aeromonas hydrophila* is a rod-shaped, motile, and gram-negative bacterium that is found in wastewater, sewage, and food [1,2,3]. It is pathogenic to fishes, amphibians, reptiles, and mammals [1,2,3,4,5,6,7,8]. Wound infection of *A. hydrophila* in humans is second most frequent after oral infection and associated with traumatic events and burns and scalds related to water and soil [1,2,3,9]. Most cases of *A. hydrophila* wound infection occur in healthy people [1,10]. In particular, *Aeromonas* sp. wound infection is reported following natural disasters, such as the tsunami and hurricane [11,12]. The wound infections due to *A. hydrophila* can progress to necrotizing soft tissue infection (NSTI) [7,8]. Necrotizing fasciitis (NF) is the most serious presentation of NSTI, an aggressive subcutaneous infection that spreads along the superficial fascia, which comprises the skin and underlying muscles, and its rapid dissemination can result in more severe disease manifestations such as sepsis [8,9,10,13,14].

Multiple factors including adhesins, hemolysin, aerolysin, nuclease, proteases, and type VI secretion system effectors are known to be involved in the pathogenesis in *A. hydrophila* infections [1,2,3,4,5,6,7,8]. Ponnusamy et al. and Fernández-Bravo et al. showed that *A. hydrophila* exotoxin A (ExoA), which has ADP ribosyl transferase activity and targets eukaryotic elongation factor-2, is a crucial factor in NF and destruction of the local tissue barrier [7,15]. However, the mechanisms involved in the rapid expansion of the necrosis and the progression to sepsis have not been clarified. Since the bacterial pathogens cause significant pathology in hosts for the colonization and proliferation, it is important to identify the mechanisms responsible for the high adaptability and proliferation ability of the pathogens in the host.

Bacterial motility is swimming activity in a liquid environment by rotating the polar and lateral flagella, and this motility enables bacteria to leave unfavorable environments for more suitable ones [16,17,18,19,20]. The flagellin that is a subunit protein of the flagellum is known as a major target as an H-antigen for recognition by the host cells through the toll-like receptor [21]. In addition, it was reported that flagellar-based motility is necessary for *A. hydrophila* adhesion during infection [22,23]. Thus, the flagellum and the flagellar-based motility is essential for the pathogenesis of *A. hydrophila* infection. However, the significance of the flagella-mediated movement inside the host body has not been studied. To rotate flagella, the flagellar motor is powered by ion gradients [16,17,18,19,24]. Stators of the motor are the transmembrane ion channel that conducts cations such as protons (H+) or sodium ions (Na+). The MotA/MotB complex is well studied in *Escherichia coli* and *Salmonella* and acts as the stator, as the channel across the cytoplasmic membrane converts H+ influx into torque by interaction with the rotor [16,17,18,19,24]. The PomA/PomB complex is a Na+-coupled stator unit generally possessed by *Vibrio* species, which live in seawater and brackish water [18,19,25,26,27,28]. *A. hydrophila* has two or more sets of motor stator proteins [29]. Since this pathogen lives in water environments with various sodium concentrations, the stators are required for adaptation and active motility. Our genome analysis revealed that RIMD111065, which is clinically isolated from patient blood, possessed two types of genes encoding MotA/MotB and PomA/PomB. These facts indicate that adaptable motor stators and motility might provide a survival advantage and could be involved in pathogenicity during the infection.

In this study, we showed that the two types of motor stators of *A. hydrophila* share homology with motor stators in other bacteria and complemented each stator in motility. Further, we employed murine infection models to investigate the pathogenesis during *A. hydrophila* infection. Here, we report that the motility of *A. hydrophila* is essential for the development of necrosis with fatal outcomes.

## 2. Materials and Methods

### 2.1. Bacteria

*A. hydrophila* clinical isolated strain RIMD111065 with spontaneous rifampicin resistance was cultured aerobically in Luria-Bertani (1% NaCl, 1% tryptone, and 0.5% yeast extract) (LB) broth or on LB agar at 37 °C. When required, the medium was supplemented with chloramphenicol (5 µg/mL) for *A. hydrophila* to maintain the pACYC plasmid. Δ*motX* is a mutant strain used as a positive control for motility [6].

### 2.2. Bioinformatics

Genome sequences were obtained from NCBI genome database (https://www.ncbi.nlm.nih.gov/genome/) and KEGG database (https://www.genome.jp/kegg/). The genome sequences of the reference strains used in this study have been deposited under the GenBank accession numbers: CP000462 (*Aeromonas hydrophila* ATCC 7966), LYXO01000024 (*Aeromonas piscicola* AH-3), U00096 (*Escherichia coli* K12), AE006468 (*Salmonella enterica* subsp. enterica serovar Typhimurium LT2), CP000626 (*Vibrio cholerae* O395), AE016795 (*Vibrio vulnificus* CMCP6), and AE014300 (*Shewanella oneidensis* MR-1). Accession number of the genome of *Aeromonas hydrophila* RIMD111065 is AP024234. Amino acid sequences were inspected by using the BLASTP network service at the NCBI and PRALINE [30].

### 2.3. Mutants Construction and Complementation

The *motA*/*motB* (hereafter *motAB*) and *pomA*/*pomB* (hereafter *pomAB*) deletion mutants were constructed by the same method for the mutant construction of *Vibrio vulnificus* [31]. Briefly, the *motAB* gene and the *pomAB* gene were amplified and cloned into the suicide vector pYAK1, retaining *sacB*. pYAK1-*motAB* KO and pYAK1-*pomAB* KO were introduced into *A. hydrophila* RIMD111065. The bacteria retaining pYAK1-*motAB* KO or pYAK1-*pomAB* KO was cultured in LB broth containing 20% sucrose following the standard *sacB*-assisted allelic exchange method. Mutants were confirmed by PCR to detect expected changes in size at the *motAB* locus or the *pomAB* locus. pYAK1-*pomAB* KO were introduced into the Δ*motAB* and also constructed the knockout strain Δ*motAB*/*pomAB.* The Δ*motAB*, the Δ*pomAB*, and Δ*motAB*/*pomAB* were complemented with the full-length *motAB*, *pomAB*, and *motAB*/*pomAB* gene carried by pACYC. The complementation strain p*motAB*, p*pomAB*, and p*motAB*/*pomAB* were cultured in LB containing 5 μg/mL chloramphenicol.

### 2.4. Cytotoxicity Assay

Cytotoxicities against HeLa cells (*n* = 6/group, MOI = 10) were determined by measuring the activity of lactate dehydrogenase (LDH) released from damaged cells at 2 h post-infection using a cytotoxicity assay kit (Cyto Tox 96; Promega KK, Tokyo, Japan).

### 2.5. Motility Assay

*A. hydrophila* was grown in LB medium with agitation (163 rpm) at 37 °C. Overnight cultures (100 μL) were inoculated into 2 mL of fresh LB medium and incubated for 12 h. Bacterial suspensions were inoculated with a sterile toothpick on LB containing 0.25% agar. Plates were incubated at 37 °C for 24 h. Motility was assessed by examining the migration of bacteria through the agar from the inoculation site.

### 2.6. Mice

Five-week-old female C57BL/6 mice were purchased from Charles River Laboratories Japan (Atsugi, Japan), bred and maintained under specific pathogen–free conditions at Kitasato University, and used for all experiments in our study.

### 2.7. Histopathological examination

*A. hydrophila* WT and Δ*motAB*/*pomAB* were grown in LB medium with agitation (163 rpm) at 37 °C. Overnight cultures (100 μL) were inoculated into 2 mL of fresh LB medium and incubated for 12 h. Bacteria were harvested, washed with PBS (pH 7.2) containing 0.1% gelatin, and resuspended in fresh LB medium. Then, PBS or 10^7^ CFU/mouse were subcutaneously inoculated in mice (*n* = 6/group). Infected mice were sacrificed at 12 h post-infection. Right caudal thighs were collected as the inoculation site, demineralized by immersing them in buffer solution containing 0.2 M EDTA-4Na and 1% formalin for 1 week, fixed in 10% buffered formalin for 1 day, embedded in paraffin, sliced into 2-µm sections, and stained with Hematoxylin-eosin. Image acquisition of the soft tissues was performed using an inverted microscope (DM2500/Leica Microsystems, Tokyo, Japan) equipped with 10×/0.40 objective lenses.

### 2.8. Evaluation of Biomarkers

*A. hydrophila* WT and Δ*motAB*/*pomAB* were grown in LB medium with agitation (163 rpm) at 37 °C. Overnight cultures (100 μL) were inoculated into 2 mL of fresh LB medium and incubated for 12 h. Bacteria were harvested, washed with PBS (pH 7.2) containing 0.1% gelatin, and resuspended in fresh LB medium. Then, PBS or 10^7^ CFU/mouse were subcutaneously inoculated in mice (*n* = 6/group). Infected mice were sacrificed at 9 h post-infection. Whole-blood samples were collected in a syringe by cardiac puncture after infection and centrifuged at 1200× *g* for 30 min, and sera were collected. Serum LDH, creatine kinase (CK), and aspartate aminotransferase (AST) concentrations were evaluated using Dimension EXL with the LM Integrated Chemistry System (Siemens, Tokyo, Japan).

### 2.9. Bacterial Counts in Muscles and Spleen

*A. hydrophila* WT and Δ*motAB*/*pomAB* were grown in LB medium with agitation (163 rpm) at 37 °C. Overnight cultures (100 μL) were inoculated into 2 mL of fresh LB medium and incubated for 12 h. Bacteria were harvested, washed with PBS (pH 7.2) containing 0.1% gelatin, and resuspended in fresh LB medium. Then, 10^7^ CFU/mouse were subcutaneously inoculated in mice (*n* = 6/group). Infected mice were sacrificed at 12 h post-infection. The muscles beneath the inoculation site and spleen were collected, suspended in cold PBS containing 0.1% gelatin, homogenized for 5 s with a lab mixer IKA EUROSTAR digital (IKA Werke, Germany; 1300 rpm), and centrifuged at 800 rpm for 5 min. The supernatants were plated at 10-fold serial dilutions in duplicate on LB agar containing 50 µg/mL rifampicin and incubated for 12 h at 37 °C. *A. hydrophila* colonies were counted, and bacterial burden was determined by calculating the number of CFU/g.

### 2.10. Mortality Rate

*A. hydrophila* WT and Δ*motAB*/*pomAB* were grown in LB medium with agitation (163 rpm) at 37 °C. Overnight cultures (100 μL) were inoculated into 2 mL of fresh LB medium and incubated for 12 h. Bacteria were harvested, washed with PBS (pH 7.2) containing 0.1% gelatin, and resuspended in fresh LB medium. Then, 10^7^ CFU/mouse were subcutaneously inoculated in mice (*n* = 6/group). Infected mice were monitored every hour for a maximum of 72 h. Mice were euthanized by inhalation of sevoflurane when exhibit hypothermia and/or are lying.

### 2.11. Statistical Analysis

Statistical analysis was performed using GraphPad Prism (GraphPad Software, CA, USA). Statistical differences between the two groups were analyzed using the Mann–Whitney *U* test. Survival curves were analyzed using the log-rank test. A *p* value less than 0.05 was considered significant, and significance values are indicated as follow: ns, not significant, *p* > 0.05; *, *p* < 0.05; **, *p* < 0.01; ***, *p* < 0.001.

## 3. Results

**Figure 1:**
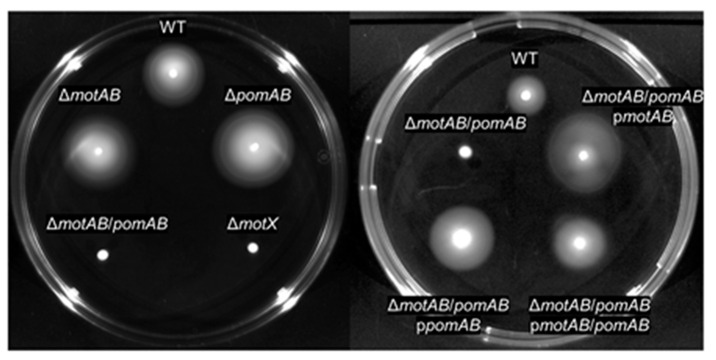
Motility phenotypes on the soft-agar plate. Bacteria were inoculated on LB plates containing 0.25% agar. Plates were incubated at 37 °C and observed at 24 h post-inoculation.

*A. hydrophila* is known to possess two or more flagellar stators. Wilhelms et al. reported that two sodium-driven motor stators are involved in flagellar rotation of *A. hydrophila* [29]. The amino acid sequences of the two stators, MotAB and pomAB, of *A. hydrophila* RIMD111065 were identical to those of *A. hydrophila* ATCC7966 (Table 1, Figures S1–S4). This indicates that the stators are conserved in *A. hydrophila* strains. Although the sequence homology of MotAB of *A. hydrophila* RIMD111065 was low compared to those of *E. coli* and *Salmonella* Typhimurium, residues for interaction with the rotor component FliG and torque generation were conserved (Table 1, Figures S1 and S2) [18,19,24]. Additionally, the homologies of MotAB of *A. hydrophila* were highest to MotAB of *Shewanella oneidensis* MR-1, which has been reported to function as a proton channel (Table 1, Figures S1–S4) [32,33]. PomA and PomB of *A. hydrophila* RIMD111065 had sequence homologies close to those of *Vibrio* species and *S. oneidensis* and had essential residues for functioning (Table 1, Figures S3 and S4) [25,26,27,28,32,33,34,35]. To determine whether the proteins play a role in motility, we created knockout strains Δ*motAB*, Δ*pomAB*, Δ*motAB*/*pomAB,* and their complemented strains. As assessed by light microscopy, WT, Δ*motAB*, and Δ*pomAB* cells displayed swimming in LB medium. Motility assay with soft agar plate showed that the motility of Δ*motAB* and Δ*pomAB* were similar to that of WT, whereas that of Δ*motAB*/*pomAB* was lacking motility, similar to non-motile strain Δ*motX* (Figure 1). In addition, motility of Δ*motAB*/*pomAB* was restored by complementing either *motAB* or *pomAB* (Figure 1). Accordingly, these results show that the two types of motor stator of *A. hydrophila* are individually sufficient to drive flagella rotation and can complement each other in motility. These results showed that both MotAB and PomAB function for motility.

**Figure 2:**
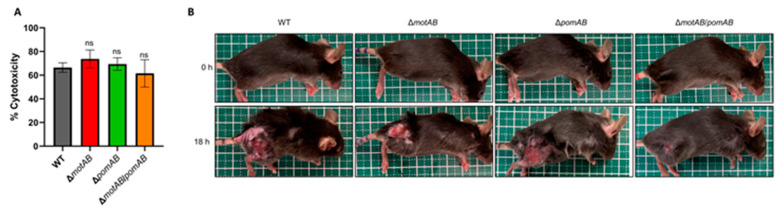
Soft tissue lesions in the *A. hydrophila* wound infection model. (**A**) Cytotoxicities against HeLa cells (*n* = 6/group, MOI = 10) were determined by measuring the activity of LDH released from damaged cells at 2 h post-infection. Error bars indicate SEM. ns, not significant (*p* > 0.05) compared with WT; Mann-Whitney *U*-test. (**B**) Mice were s.c.-inoculated with WT, Δ*motAB*, Δ*pomAB*, and Δ*motAB*/*pomAB.* Pictures show infected mice at 0 h and 18 h post-infection.

We investigated the effect of motility on cytotoxicity and pathogenicity of *A. hydrophila*. LDH release assay revealed no significant difference in cytotoxicity against HeLa cells between all mutants and WT. This result showed that the loss of motor stators and motility does not affect the in vitro cytotoxicity (Figure 2A). Next, 10^7^ CFU of *A. hydrophila* RIMD111065 WT, Δ*motAB*, Δ*pomAB*, and Δ*motAB*/*pomAB* were subcutaneously inoculated into mice. Careful observation showed that mice infected with the WT, Δ*motAB*, and Δ*pomAB* had extensive hair loss, swelling, and lesions (Figure 2B). On the other hand, the mice infected with the non-motile strain Δ*motAB*/*pomAB* showed very localized hair loss and lesions (Figure 2B). These findings suggest that the extent of pathogenesis in soft tissue is determined by the motility of *A. hydrophila*.

**Figure 3:**
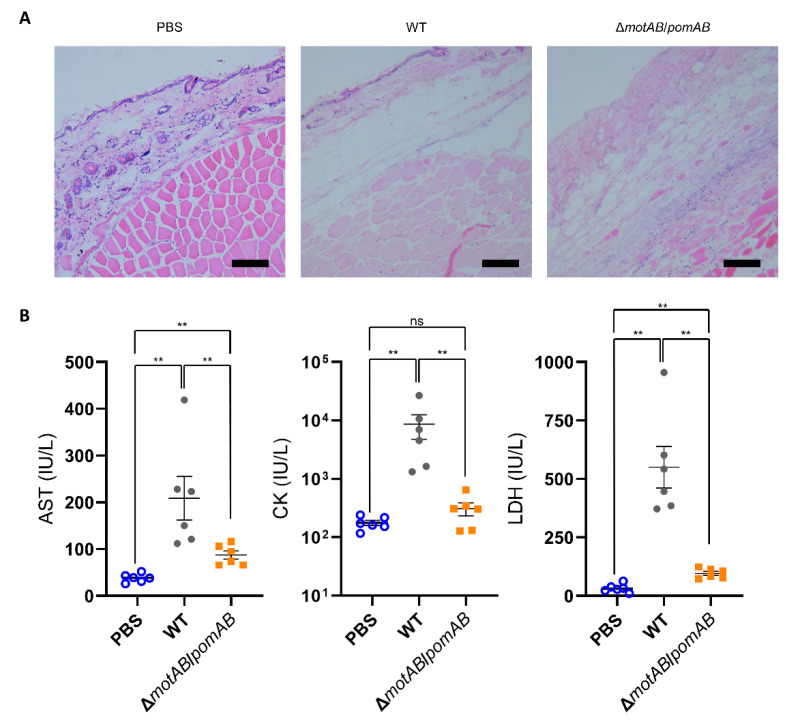
Mice were s.c.-inoculated with PBS, WT, and Δ*motAB*/*pomAB.* (**A**) Right caudal thighs were collected from mice at 12 h post-inoculation and stained with Hematoxylin-eosin. Scale bars, 10 μm. (**B**) The levels of AST, CK, and LDH in the sera of mice were measured at 9 h post-infection. Each symbol represents an individual mouse (*n* = 6/group). Error bars indicate SEM. ns, not significant (*p* > 0.05), **, *p* < 0.01; Mann-Whitney *U*-test.

To evaluate severity of the soft tissue lesions, we performed histopathological analysis and serum biochemical tests of CK, AST, and LDH. Eosin-stained fascia and muscle were clearly observed in the thighs of PBS-inoculated mice by Hematoxylin-eosin stain (Figure 3A). However, fascia and muscles were collapsed and indistinct staining with eosin in the thighs of mice infected with WT and Δ*motAB*/*pomAB* (Figure 3A). The levels of all enzymes CK, AST, and LDH in mice infected with WT were higher than those in mice infected with non-motile strain Δ*motAB*/*pomAB* (Figure 3B). The levels of AST and LDH in mice infected with Δ*motAB*/*pomAB* were higher than those in mice inoculated with PBS (Figure 3B). These results demonstrated that the motility contributes to the severity and rapid spread of soft tissue lesions during the *A. hydrophila* infection.

*A. hydrophila* rapidly proliferates in mammalian hosts and then causes fulminant diseases such as NF and bacteremia within a short time period [7,8,9,10,13,14]. To determine the effect of motility on the proliferation of *A. hydrophila* in a murine wound infection model, we collected bacteria from muscles and spleen at 6 h and 12 h after infection with *A. hydrophila* RIMD111065 WT and non-motile strain Δ*motAB*/*pomAB*. The number of bacteria collected from the muscle of mice infected with WT was increased from 6 h to 12 h (Figure 3A). Similarly, Δ*motAB*/*pomAB* were detected from the muscle at 6 h post-infection, and the number of bacteria was increased at 12 h post-infection (Figure 3A). In infection with both strains, the bacteria were detected from the spleen at 6 h post-infection, and there was no increase in the number of bacteria detected from the spleen at 12 h post-infection (Figure 3B). These results indicate that *A. hydrophila* RIMD111065 is not dependent on its motility to achieve proliferation at the local infection site and invasion of the systemic circulation resulting in bacteremia.

**Figure 4:**
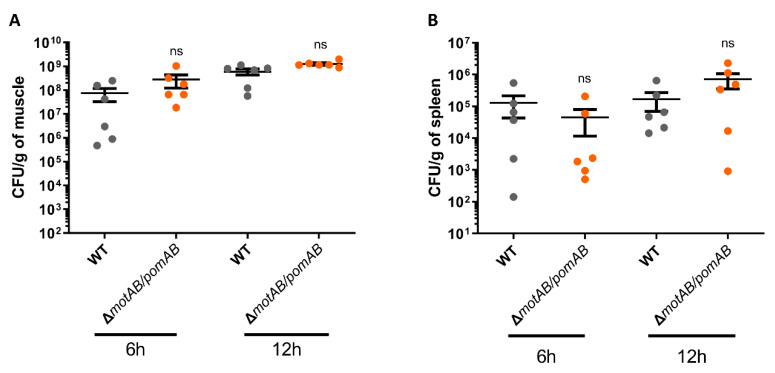
Bacterial proliferation in the host independent from motility. Bacterial burdens in the muscle tissue (**A**) and spleen (**B**) of mice s.c.-inoculated with WT and Δ*motAB*/*pomAB* calculated as CFU/g at 6 h and12 h post-infection. Each symbol represents an individual mouse (*n* = 6/group). Error bars indicate SEM. ns, not significant (*p* > 0.05) compared with WT; Mann–Whitney *U*-test.

To determine the motility-dependent lethality in the murine model, we performed a survival curve analysis for mice infected with WT and non-motile strain Δ*motAB*/*pomAB*. All mice s.c.-inoculated with 10^7^ CFU of WT died (Figure 4). The mice s.c.-inoculated with Δ*motAB*/*pomAB* also died, but their survival time was significantly prolonged (Figure 4). Taken together, the motility of *A. hydrophila* is essential for progression to a fatal condition within a short time in mammalian hosts.

## 4. Discussion

NF caused by *A. hydrophila* progresses rapidly within a short time period [7,8,9,10]. This makes it difficult to treat patients and leads to significant sequelae or fatal outcomes. However, there are few studies on the mechanisms for the rapid expansion of necrotic lesions. In our wound infection model, the necrotic lesions caused by WT expanded rapidly, but the necrotic lesions caused by the non-motile strain were very localized (Figure 2B and Figure 3A). This study showed here for the first time that the motility of *A. hydrophila* is a crucial virulence factor associated with rapid expansion of necrotic lesions.

**Figure 5:**
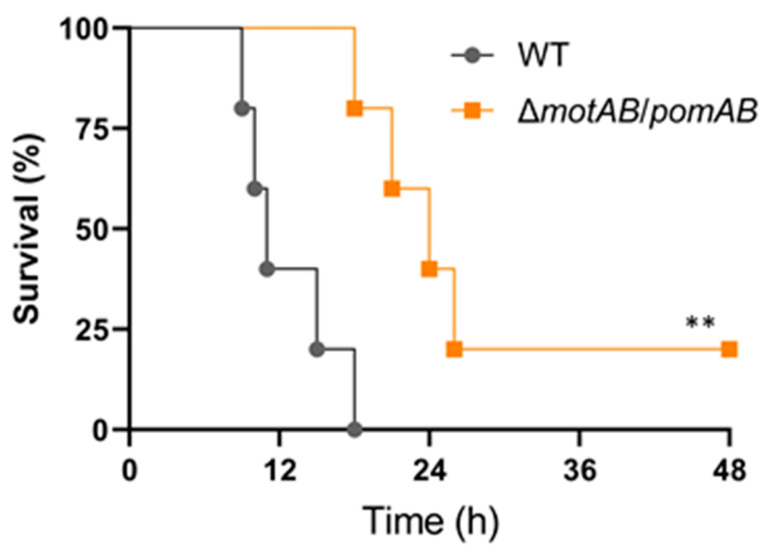
Motility-dependent lethality in the *A. hydrophila* infection model. Kaplan–Meier survival curves for mice inoculated s.c. with WT and Δ*motAB*/*pomAB* and monitored for 72 h. Each symbol represents an individual mouse (*n* = 6/group). **, *p* < 0.01; log-rank test.

Most cases of *A. hydrophila* infections are known as a polymicrobial or mixed infection with different strains of *A. hydrophila* (same species) and other bacterial pathogens, such as *Campylobacter*, *Salmonella*, and *Staphylococcus aureus* [1,2,9,10,11]. The infections frequently become severe in immunocompromised hosts such as liver disease [1,2,9]. However, RIMD111065 showed pathogenicity and lethality in healthy mice by monomicrobial infection (Figure 2B, Figure 3 and Figure 5). These results indicated that this strain has a mechanism to evade the immune system at the site of infection or in the internal organs of a healthy host, not depending on other pathogens. Additionally, since the non-motile strain was detected from the local infection site and the spleen similar to the WT, this study showed that the evasion mechanism does not depend on the bacterial motility.

*A. hydrophila* is a motile pathogen known to be distributed in various aquatic environments and infects a wide variety of hosts, including fish and mammals [1,2,3,4,5,6,7,8]. Motility is generally affected by the secretion of toxins, the quorum-sensing system, and/or the environment [4,7,8,16,17,18,19,36,37,38]. As an example of how toxin secretion affects motility, Ponnusamy et al. showed that the motility of *A. hydrophila* was significantly increased when *exoA* was deleted [7]. Quorum sensing is necessary for efficient proliferation both in the environment and in the host and may switch between motile state and non-motile state, depending on the bacterial density [37,38]. Kozlova et al. showed that quorum-sensing mutations in *A. hydrophila* reduce motility [37]. Jahid et al. showed that differences in salt concentration in the environment affect quorum-sensing and motility [4]. They also showed that *A. hydrophila* proliferates and is motile in a wide range of salt concentrations from 0% to 3% [4]. This adaptability is thought to be due to the two types of motor stator possessed by *A. hydrophila* as shown in this study. We also demonstrated that *A. hydrophila* has mutually complementary MotAB and PomAB (Figure 1). Flagellar systems with the two types of stators have only been found in a few bacteria such as *S. oneidensis* and *V. alginolyticus* [25,32,33]. The adaptability of *A. hydrophila* contributes to the proliferation and the pathogenicity in the host.

Various toxins are involved in the pathogenicity of *A. hydrophila* infection [5,6,7,8]. All strains used in this study caused cytotoxicity (Figure 2A). This result showed that defects in the two types of stators did not affect toxin secretion for cytotoxicity. Aerolysin is well known cytotoxic enterotoxin of *A. hydrophila* [8,39,40,41]. It is secreted by the type II secretion system and also known to affect lethality in mice, although its role in wound infection is unknown. RIMD111065 has a gene encoding Aerolysin, and this toxin may contribute to the cytotoxicity and lethality. A toxin ExoA is reported as a crucial virulence factor involved in the onset of the NF in *A. hydrophila* infection [7,11]. However, RIMD111065 does not have a gene encoding ExoA. The present study revealed that this strain caused necrotic lesions (Figure 2B and Figure 3A). These facts strongly suggest that RIMD111065 may have a novel necrosis factor not dependent on ExoA. In addition, its motility contributed to aggravation. Thus, the necrosis-related factors will function only in the local site where the *A. hydrophila* proliferates after spreading through soft tissues by its motility.

The effects of motility on bacterial pathogenicity have been reported in a study on *Vibrio vulnificus* wound infection [31,42,43]. *Vibrio vulnificus* is also one of the causative agents of NSTI, and clinical syndromes resulting from infection with *A. hydrophila* resemble those due to *Vibrio vulnificus*. A motility-deficient mutant of *V. vulnificus* had reduced proliferation ability and pathogenicity at the site of infection [31]. However, the proliferation ability of *A. hydrophila* was not affected by lack of motility as shown in this study. *V. vulnificus* requires motility to expand necrosis from the superficial layer to the deep layer in the muscle, while *A. hydrophila* required motility to expand necrosis in the skin. These findings provide useful evidence that the motility-dependent pathogenic mechanisms of *A. hydrophila* and *V. vulnificus* have an effect on clinical symptoms.

Our results also indicate that *A. hydrophila* has an ability to invade the systemic circulation and then proliferate independent of motility. Romero et al. reported that non-motile *A. hydrophila* AH-1:: motX can proliferate without motility in models of fish and intraperitoneally infected mice [6]. Their analysis with strain lacking polar flagella showed that *A. hydrophila* require flagellar-based adhesion to proliferate in the circulation. In addition, our study shows that the extent of necrosis based of flagellar motility influences the survival time of mice. This is the first study to report motility as one of the critical factors for the evolution of *A. hydrophila* NSTI.

## Figures and Tables

**Table 1:** The homology scores and key residues of stator proteins in RIMD111065 and others.

Protein	Organism	Strain	Length	E Value	Identities	Positives	Interaction with FliG	Ion Channel Pore	Ion Binding	Torque Generation
MotA	*A. hydrophila*	RIMD111065	252	-	-	-	Arg86 and Glu94	-	-	Pro150 and Pro199
	*A. hydrophila*	ATCC7966	252	0.00E+00	252/252(100%)	252/252(100%)	Arg86 and Glu94	-	-	Pro150 and Pro199
	*A. piscicola*	AH-3	252	9E−177	234/251(93%)	240/251(95%)	Arg86 and Glu94	Phe183	Asn194	Pro150 and Pro199
	*E. coli*	K12	295	4.00E−16	55/210(26%)	93/210(44%)	Arg90 and Glu98	Met206	Tyr217	Pro173 and Pro222
	*S.* *typhimurium*	LT2	295	3.00E−17	59/211(28%)	96/211(45%)	Arg90 and Glu98	Met206	Tyr217	Pro173 and Pro222
	*S. oneidensis*	MR-1	243	3.00E−134	174/242(72%)	207/242(85%)	Arg86 and Glu94	-	-	Pro150 and Pro199
MotB	*A. hydrophila*	RIMD111065	291	-	-	-	Asp21	Ala29	Asp21	-
	*A. hydrophila*	ATCC7966	291	0	291/291(100%)	291/291(100%)	Asp21	Ala29	Asp21	
	*A. piscicola*	AH-3	291	0	280/291(96%)	285/291(97%)	Asp21	Ala29	Asp21	
	*E. coli*	K12	308	3.00E−16	73/296(25%)	135/296(45%)	Asp32	Ala39	Asp32	
	*S.* *typhimurium*	LT2	309	3.00E−15	72/293(25%)	124/293(42%)	Asp33	Ala40	Asp33	
	*S. oneidensis*	MR-1	275	1.00E−95	133/289(46%)	196/289(67%)	Asp21	Ala28	Asp21	
PomA	*A. hydrophila*	RIMD111065	252	-	-	-	Arg88 and Glu96	Leu183	Asn194	Pro151 and Pro199
	*A. hydrophila*	ATCC7966	252	0	252/252(100%)	252/252(100%)	Arg88 and Glu96	Leu183	Asn194	Pro151 and Pro199
	*A. piscicola*	AH-3	245	8E−35	69/241(29%)	127/241(52%)	Arg85 and Glu93	-	Asn193	pro150 and pro198
	*V. cholerae*	O395	254	1.00E−116	164/247(66%)	199/247(80%)	Arg88 and Glu96	Leu183	Asn194	Pro151 and Pro199
	*V.* *vulnificus*	CMCP6	253	3.00E−122	163/247(66%)	200/247(80%)	Arg88 and Glu96	Leu183	Asn194	Pro151 and Pro199
	*S. oneidensis*	MR-1	255	6.00E−121	159/251(63%)	201/251(80%)	Arg88 and Glu96	Leu183	Asn194	Pro151 and Pro199
PomB	*A. hydrophila*	RIMD111065	299	-	-	-	Asp20	Cys27	Asp20	Phe18
	*A. hydrophila*	ATCC7966	299	0	299/299(100%)	299/299(100%)	Asp20	Cys27	Asp20	Phe18
	*A. piscicola*	AH-3	303	2E−22	70/305(23%)	124/305(40%)	Asp24	-	Asp24	-
	*V. cholerae*	O395	318	1.00E−134	182/302(60%)	231/302(76%)	Asp23	Cys30	Asp23	Phe21
	*V.* *vulnificus*	CMCP6	318	4.00E−137	187/308(61%)	234/308(75%)	Asp24	Cys31	Asp24	Phe22
	*S. oneidensis*	MR-1	308	9.00E−132	182/302(60%)	222/302(73%)	Asp20	Cys27	Asp20	Phe18

## Data Availability

Sequence data that support the findings of this study have been deposited in DDBJ Sequence Database and DRA (https://www.ddbj.nig.ac.jp/index.html). Accession numbers are available in Materials and Methods. The complete genome sequence for *A. hydrophila* RIMD111065 can be found under bioproject ID PRJDB10897, specifically, complete chromosome (GenBank accession: AP024234).

## Supplementary Materials

The following are available online at https://www.mdpi.com/2076-2607/9/1/10/s1, Figure S1: Protein sequence of MotA were aligned by manually PRALINE program, Figure S2: Protein sequence of MotB were aligned by manually PRALINE program, Figure S3: Protein sequence of PomA were aligned by manually PRALINE program, Figure S4: Protein sequence of PomB were aligned by manually PRALINE program.
