# Supplementary material for: Expansion of Necrosis Depending on Hybrid Motor-Driven Motility of Aeromonas hydrophila in a Murine Wound Infection Model
Source: Microorganisms. 2020 Dec 22;9(1):10. doi: 10.3390/microorganisms9010010 (PMC7822177; doi:10.3390/microorganisms9010010)
Supplement: Supplementary file 1 [file microorganisms-09-00010-s001.pdf]

Expansion of Necrosis Depending on Hybrid Motor-Driven Motility of *Aeromonas hydrophila* in a Murine Wound Infection Model

Kohei Yamazaki [1], Takashige Kashimoto [1,*], Ayuha Niwano [1], Moeko Yamasaki [1], Mayu Nomura [1], Yukihiro Akeda [2] and Shunji Ueno [1]

[1] Laboratory of Veterinary Public Health, School of Veterinary Medicine, Kitasato University, 23-35-1, Higashi, Towada Aomori 034-8628, Japan.

[2] Division of Infection Control and Prevention, Osaka University Hospital, 2-2, Yamadaoka, Suita. Osaka 565-0871, Japan.

* Correspondence: kashimot@vmas.kitasato-u.ac.jp Laboratory of Veterinary Public Health, School of Veterinary Medicine, Kitasato University, 23-35-1, Higashi, Towada Aomori 034-8628, Japan; Tel.: +81-176-23-4371 (ext.443)

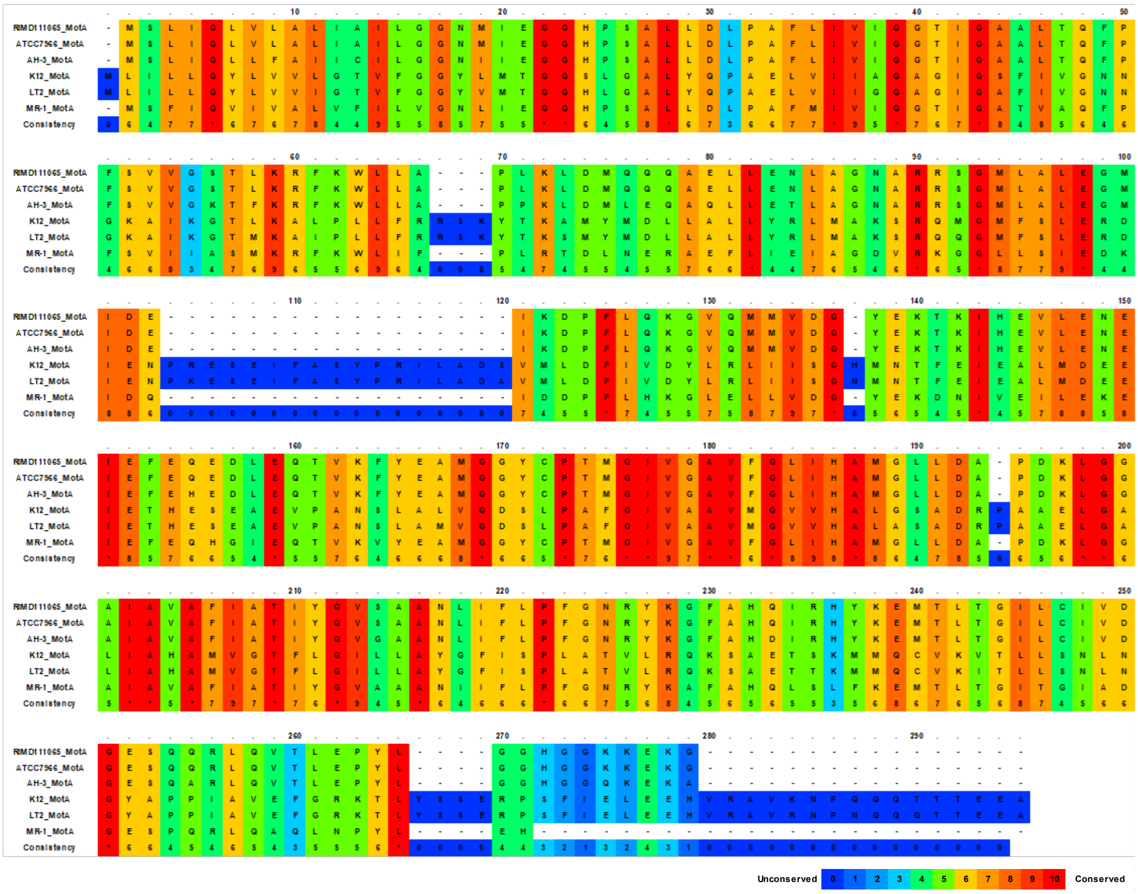

Figure S1. Protein sequence of MotA were aligned by manually PRALINE program. The conservation between the indicated sequences was scored by PRALINE.

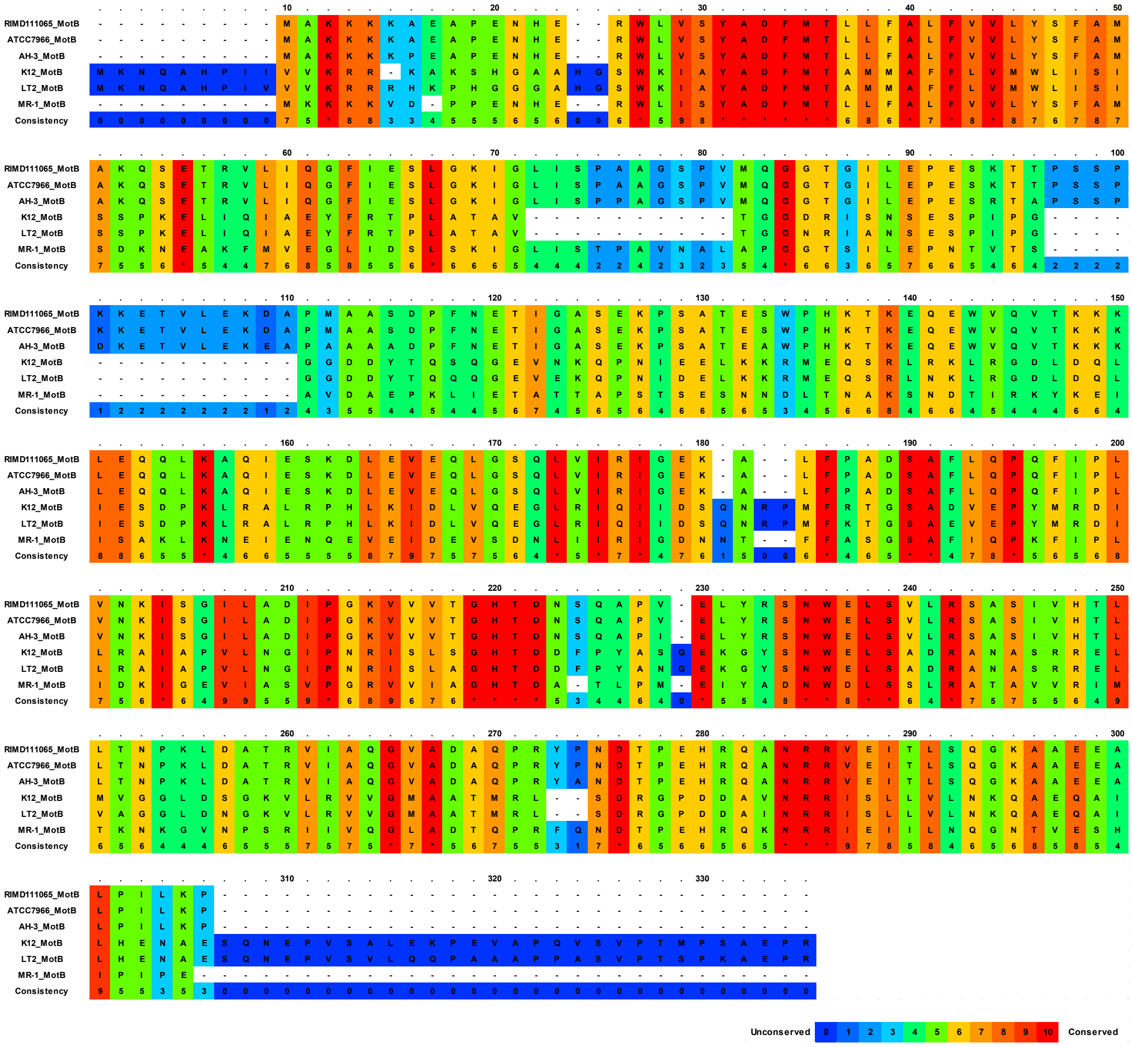

Figure S2. Protein sequence of MotB were aligned by manually PRALINE program. The conservation between the indicated sequences was scored by PRALINE.

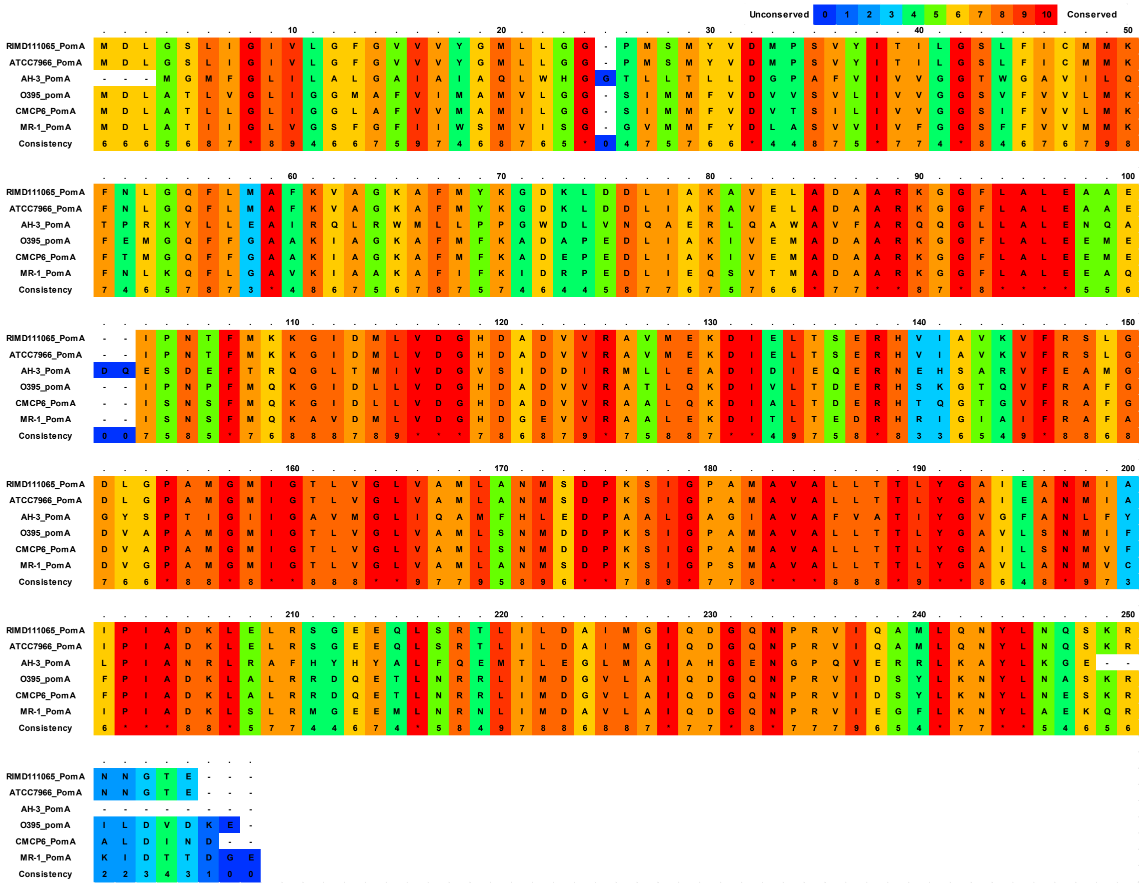

Figure S3. Protein sequence of PomA were aligned by manually PRALINE program. The conservation between the indicated sequences was scored by PRALINE.

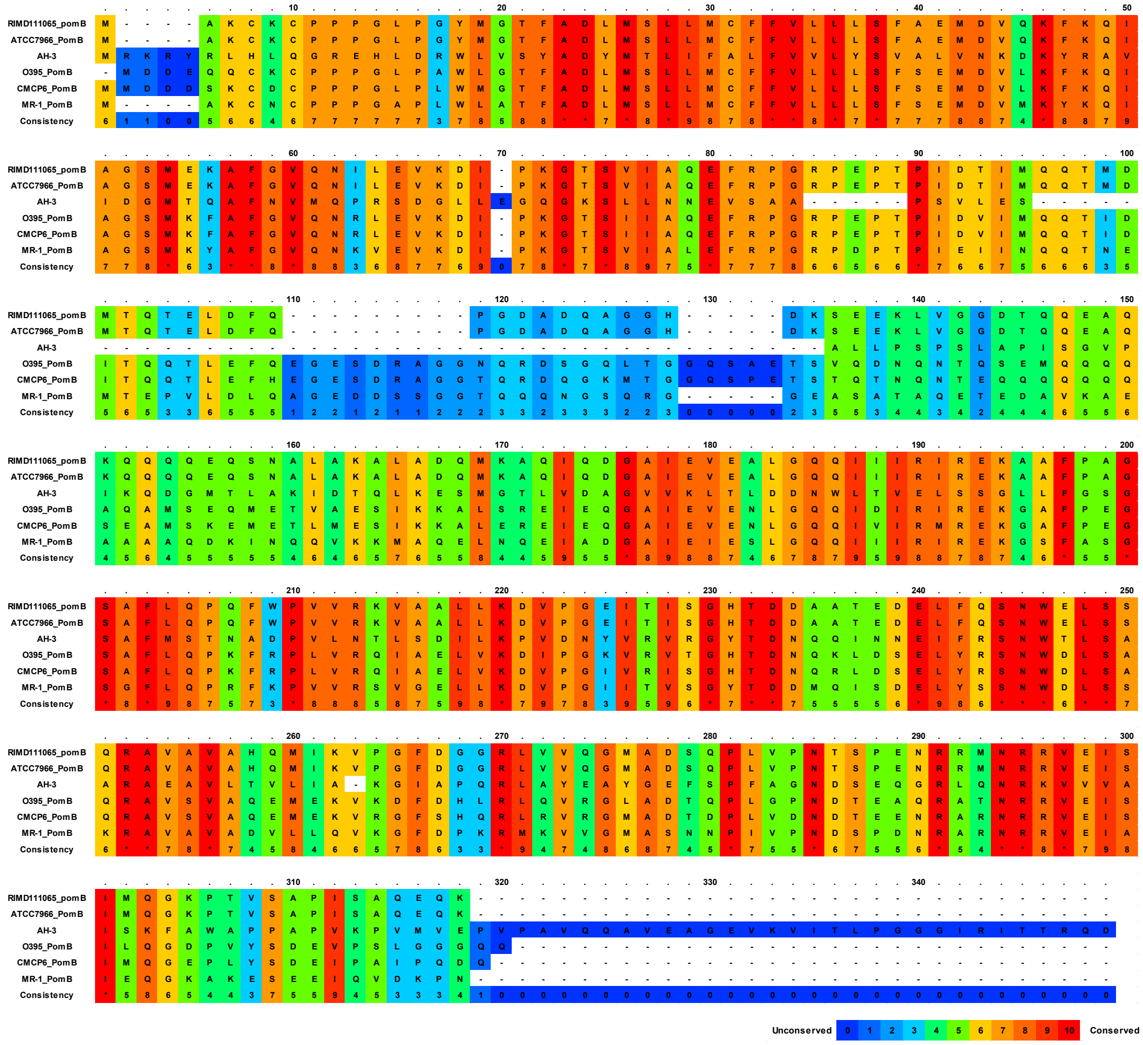

Figure S4. Protein sequence of PomB were aligned by manually PRALINE program. The conservation between the indicated sequences was scored by PRALINE.
